# *In Silico* Analysis of Six Known *Leishmania major* Antigens and *In Vitro* Evaluation of Specific Epitopes Eliciting HLA-A2 Restricted CD8 T Cell Response

**DOI:** 10.1371/journal.pntd.0001295

**Published:** 2011-09-06

**Authors:** Negar Seyed, Farnaz Zahedifard, Shima Safaiyan, Elham Gholami, Fatemeh Doustdari, Kayhan Azadmanesh, Maryam Mirzaei, Nasir Saeedi Eslami, Akbar Khadem Sadegh, Ali Eslami far, Iraj Sharifi, Sima Rafati

**Affiliations:** 1 Molecular Immunology and Vaccine Research Lab, Pasteur Institute of Iran, Tehran, Iran; 2 Department of Virology, Pasteur Institute of Iran, Tehran, Iran; 3 Health Care Center, Chaheshk, Mashhad, Iran; 4 National Cell Bank of Iran, Pasteur Institute of Iran, Tehran, Iran; 5 Department of Electron Microscopy and Clinical Research, Pasteur Institute of Iran, Tehran, Iran; 6 School of Medicine, Leishmaniasis Research Center, Kerman University of Medical Sciences, Kerman, Iran; Institut Pasteur de Tunis, Tunisia

## Abstract

**Background:**

As a potent CD8^+^ T cell activator, peptide vaccine has found its way in vaccine development against intracellular infections and cancer, but not against leishmaniasis. The first step toward a peptide vaccine is epitope mapping of different proteins according to the most frequent HLA types in a population.

**Methods and Findings:**

Six *Leishmania (L.) major*-related candidate antigens (CPB,CPC,LmsTI-1,TSA,LeIF and LPG-3) were screened for potential CD8^+^ T cell activating 9-mer epitopes presented by HLA-A*0201 (the most frequent HLA-A allele). Online software including SYFPEITHI, BIMAS, EpiJen, Rankpep, nHLApred, NetCTL and Multipred were used. Peptides were selected only if predicted by almost all programs, according to their predictive scores. Pan-A2 presentation of selected peptides was confirmed by NetMHCPan1.1. Selected peptides were pooled in four peptide groups and the immunogenicity was evaluated by *in vitro* stimulation and intracellular cytokine assay of PBMCs from HLA-A2^+^ individuals recovered from *L. major*. HLA-A2^−^ individuals recovered from *L. major* and HLA-A2^+^ healthy donors were included as control groups. Individual response of HLA-A2^+^ recovered volunteers as percent of CD8^+^/IFN-γ^+^ T cells after *in vitro* stimulation against peptide pools II and IV was notably higher than that of HLA-A2^−^ recovered individuals. Based on cutoff scores calculated from the response of HLA-A2^−^ recovered individuals, 31.6% and 13.3% of HLA-A2^+^ recovered persons responded above cutoff in pools II and IV, respectively. ELISpot and ELISA results confirmed flow cytometry analysis. The response of HLA-A2^−^ recovered individuals against peptide pools I and III was detected similar and even higher than HLA-A2^+^ recovered individuals.

**Conclusion:**

Using *in silico* prediction we demonstrated specific response to LmsTI-1 (pool II) and LPG-3- (pool IV) related peptides specifically presented in HLA-A*0201 context. This is among the very few reports mapping *L. major* epitopes for human HLA types. Studies like this will speed up polytope vaccine idea towards leishmaniasis.

## Author Summary

Leishmaniasis is currently a serious health as well as economic problem in underdeveloped and developing countries in Africa, Asia, the Near and Middle East, Central and South America and the Mediterranean region. Cutaneous leishmaniasis is highly endemic in Iran, remarkably in Isfahan, Fars, Khorasan, Khozestan and Kerman provinces. Since effective prevention is not available and current curative therapy is expensive, often poorly tolerated and not always effective, alternative therapies including vaccination against leishmaniasis are of priority to overcome the problem. Although Th1 dominant response is so far considered as a pre-requisite for the immune system to overcome the infection, CD8^+^ T cell response could also be considered as a potent arm of immune system fighting against intracellular *Leishmania*. Polytope vaccine strategy may open up a new way in vaccine design against leishmaniasis, since they act as a potent tool to stimulate multi-CD8 T cell responses. Clearly there is a substantial need to evaluate the promising epitopes from different proteins of *Leishmania* parasite species. Some new immunoinformatic tools are now available to speed up this process, and we have shown here that *in silico* prediction can effectively evaluate HLA class I-restricted epitopes out of *Leishmania* proteins.

## Introduction

Leishmaniasis is a parasitic disease found in tropical and subtropical countries and also in southern Europe. It is caused by infection with *Leishmania* parasites, which are spread by the bite of infected sand flies. There are several different forms of leishmaniasis in people; the most common are cutaneous leishmaniasis (CL), which causes skin sores, and visceral leishmaniasis (VL), which affects some of the internal organs of the body (http://www.cdc.gov/NCIDOD/DPD/parasites/leishmania/). Although CL does not end in death, many problems are faced due to long-lasting lesions, cosmetic problems, high expenses of treatment, side effects of existing drugs and drug resistance. Despite the huge number of publications on different vaccination strategies, there is yet no protective vaccine in routine use for humans. Current control relies on chemotherapy to alleviate the disease [1]–[3] and on vector control to reduce transmission [4].

It has been a consensus for a long time that a Th1 dominant response instead of Th2 promotes IFN-γ production, which activates macrophages to kill parasites via nitric oxide production and induces lesion healing and control of the parasite burden [5]–[9]. Based on this theory, different vaccination strategies have been examined so far including leishmanization [10], killed parasites [11], live attenuated parasites [12], subunit vaccines including recombinant or native proteins of different stages of parasite life cycle and DNA vaccines [13]–[18], dendritic cell-based vaccines [19], [20], salivary antigen-based vaccines [21], [22] and non-pathogenic parasite-based vaccines [23]. Although many of these strategies have shown promising results in mice [24]–[27] and dogs [28]–[30], none of them has entered human trials except for Leish-F1 (a recombinant fusion protein of LmsTI-1, TSA and LeIF) with reported phase I and II clinical trials [31], [32].

On the other hand, CD8^+^ T cells as a potent arm of adaptive immunity have drawn attention in controlling leishmaniasis, since growing evidence has proved their participation in immune response against different *Leishmania* species studied in experimental models and human. IFN-γ production by these cells diverts a transient Th2 response at the very beginning to Th1 [33] and modulates the IFN-γ production by CD4^+^ T cells late after [34], [35], which ends in disease control at primary infection of C57BL/6 mice. Muller *et al.* also showed an elevated IFN-γ production at secondary infection of immune mice to *L. major* due to CD8^+^ T cells [36], [37]. These responses are also associated with the cure of CL in human [38], [39]. A major concern about CD8^+^ T cell activation refers back to cytotoxic activity of these cells. Despite cytokine production which is thoroughly analyzed [40], the role of cytotoxic activity in protection is still under investigation. Cytotoxic activity has been shown at the site of infection [41] concomitant with killing of parasite at CL cases [42], [43], but Diffuse Cutaneous leishmaniasis and Muco-cutaneous leishmaniasis stay at two extremities of cytotoxic activity; the former is associated with exhausted cytotoxicity [44] and the later is associated with exaggerated activity and destructive effects [45] compared to CL cases.

Today, there are many reports about different *Leishmania* antigens eliciting CD8^+^ T cell responses such as P8 and gp46 [46], Kmp11 [47], CPB [48], nucleosomal histones [49], LmaCIN [50], LmsTI-1 and TSA [51] and A2 [52]. The concept that CD8^+^ T lymphocytes could be important in protection and long-lasting resistance to infection has opened up a new strategy in *Leishmania* vaccine design known as “Polytope Vaccine” [53]. This strategy is a new hope in vaccine field against leishmaniasis for upcoming future [54]. Polytope vaccines are mainly designed as DNA constructs encompassing nucleotide sequence of multiple epitopes in tandem. This strategy faces challenges such as best peptide arrangement in tandem [55], [56] or flanking sequences of each peptide [57]–[59] to make the chance of being chopped into right peptides. However, it possesses some extraordinary advantages over other subunit vaccines, especially the ability to direct the immune system towards multi-epitope CD8^+^ T cell responses [60], [61].

The first step toward a polytope vaccine idea is the identification of HLA class I-restricted epitopes that are naturally processed and presented in the context of HLA class I and potentially activate CD8^+^ T cells. Since there are very few reported epitopes specific for *Leishmania major* (the main cause of CL in Iran) proteins, we took advantage of the potential of immunoinformatic tools to screen for *L. major*-specific epitopes from six known proteins that could be presented via HLA-A2 (the most prevalent HLA supertype in Iranian population). Studies like this will help to speed up polytope idea towards leishmaniasis, since more and more epitopes are required to be included in a vaccine if pilot studies prove this strategy protective against cutaneous leishmaniasis.

## Materials and Methods

### Protein selection

Antigens selected for this study included: CPB, CPC, LmsTI-1, TSA, LeIF and LPG-3. The full sequences of the proteins were extracted from GeneDB data on *L. major* strain MHOM/IL/80/Friedlin summarized in Table 1. These proteins are already defined as candidate antigens and selected based on high expression in amastigote stage and/or potential to stimulate CD8^+^ T cell responses.

**Table 1:** *L. major* specific proteins used as candidate antigens for 9-mer epitope screening.

Abbreviations	Names	Accession number^a^
CPB	Cathepsin L-like Protease or Type I Cysteine Proteinase	LmjF08.1080
CPC	Cathepsin B-like Protease or Type III Cysteine Peptidase	LmjF29.0820
LmsTI-1	*L .major* Stress Indussible Protein	LmjF08.1110
TSA	Thiol-Specific-Antioxidant	LmjF15.1100
LeIF	Elongation Initiation Factor 2 Alpha Subunit	LmjF03.0980
LPG-3	Lipophosphoglycan Biosynthetic Protein	LmjF29.0760

a The full sequences of the proteins were extracted from GeneDB data of *L. major* strain MHOM/IL/80/Fredlin.

SignalP (http://www.cbs.dtu.dk/services/SignalP) analysis confirms that CPB and CPC are secretory proteins, LPG-3 is a membrane-associated protein and the other three proteins are non-secretory. The sequence of the protein analyzed by different software included the signal peptide sequence.

### Peptide prediction

To map the promising epitopes from six known proteins of *L. major*, we focused on HLA-A*0201 because it is the most prevalent allele in white population and is the most extensively studied HLA class I allele. Data from dbMHC (http://www.ncbi.nlm.nih.gov/dbMHC) shows the frequency of this allele about 25% in south-west Asia. Low-resolution molecular HLA typing in Iranian population has also confirmed the high frequency of HLA-A2 alleles compared with other populations of the world [62]. We focused on 9-mer long peptides since HLA class I binds 9-mers more frequently than 8, 10 or 11-mer peptides. In the first step of analysis, protein sequences were screened individually for best binding epitopes with the most common online algorithms: SYFPEITHI [63] and BIMAS [64]. The cutoff score was adjusted above 20 for SYFPEITHI and 100 for BIMAS (with some exceptions for BIMAS score if necessary). Peptides matching both criteria were selected for a second step of analysis. For this, protein sequences were analyzed with five different algorithms for HLA-A*0201 including EpiJen [65], Rankpep [66], nHLApred [67], NetCTL [68] and Multipred [69]. The accuracy of all prediction algorithms was set above 80–85% based on the thresholds used (Table 2). Selected peptides through this process were then analyzed by NetMHCpan1.1 [70] to check for the possibility of binding to different alleles of HLA-A2 supertype specified as HLA-A*0202-A*0206 and A*0209 (Table 3). These alleles are the most prevalent ones in South West Asian populations as recorded by dbMHC. Peptides with 100% identity to mice or human proteins were excluded from the selected list based on BLASTP analysis and replaced with others passing the criteria of whole sieving process if possible. Peptides were synthesized with more than 90% purity by Biosynthesis Company (Lewisville, TX, USA). Lyophilized powder was dissolved in Dimethyl Sulphoxide (DMSO-Sigma, Germany) and stored aliquoted at −80°C till use.

**Table 2:** Characteristics of *in silico* predicted *L. major* specific CD8^+^ T cell 9-mer peptides restricted to HLA-A*0201 allele.

			scores						
Protein	Position^a^	Peptide sequence	SYFPEITHI	BIMAS	EpiJen^b^	RANKpep^c^/Proteasome^d^	nHLAPred^e^	NetCTL^f^	Multipred^g^
CBP	192–200	LMLQAFEWV	22	1617	+	72/−	1	1.255	MB
	285–293	QLNHGVLLV	28	159	+	73/+	1	1.055	MB
	330–338	LLTGYPVSV	28	118	+	91/−	1	1.284	MB
CPC	281–289	FLGGHAVKL	27	98	+	73/+	0.97	1.097	MB
	18–26	LLATTVSGL	29	83	+	90/+	1	1,137	MB
LmSTI-1	146–154	LLMLQPDYV	23	1179	+	68/+	1	1.027	MB
	445–453	ALQAYDEGL	24	10	+	63/+	0.93	1.218	MB
	31–39	QLDEQNSVL	22	14	+	64/+	1	0.791	MB
	170–178	YMEDQRFAL	21	108	+	72/+	0.99	1,102	MB
TSA	158–166	RLLEAFQFV	24	11025	+	87/+	0.99	1.374	MB
	104–112	MLADKTKSI	25	73	+	92/+	1	1.194	MB
LeIF	86–94	VLLEKATIL	26	225	+	86/+	1	1.05	MB
	317–325	KVLTLFAVE	22	149	+	81/+	0.61	1.225	MB
	152–160	VVWVKITQV	21	197	+	68/+	−	0.934	MB
LPG-3	14–22	LLLLGSVTV	30	437	+	86/+	1	1.023	MB
	164–172	FLVGDRVRV	25	319	+	80/+	1	1.191	MB
	41–49	MLDILVNSL	28	33	+	76/+	1	1.142	MB
	655–663	MTAERVLEV	25	15	+	74/+	1	1.181	HB

a Amino acid position as in the protein sequence.

b Threshold set on 5% (percent of whole protein peptides that should be tested).

c Specific binding threshold set on 2% (percent of whole protein peptides that should be tested).

d Proteasomal cleavage.

e Cut off score set on 0.5 (Threshold of binding).

f Threshold for epitope selection set on >0.75 (Threshold of binding).

g Promiscuous epitope predicted as moderate or high binder.

**Table 3:** HLA-A2 super-type binding possibility of selected peptides predicted by NetMHCPan1.1.

			HLA-A2 super-type						
Peptide pool	Protein	Position	A0201	A0202	A0203	A0204	A0205	A0206	A0209
Pool I	CBP	192–200	SB*	SB	SB	SB	SB	SB	SB
		285–293	WB**	SB	SB	WB	WB	WB	WB
		330–338	SB	SB	SB	SB	SB	SB	SB
	CPC	281–289	SB	SB	SB	WB	SB	SB	SB
		18–26	SB	SB	SB	WB	SB	WB	SB
Pool II	LmSTI-1	146–154	SB	SB	SB	SB	SB	SB	SB
		445–453	WB	SB	WB	WB	WB	WB	WB
		31–39	-	WB	-	-	-	-	-
		170–178	SB	SB	SB	WB	SB	SB	SB
Pool III	TSA	158–166	SB	SB	SB	SB	SB	SB	SB
		104–112	SB	SB	SB	WB	SB	SB	SB
	LeIF	86–94	WB	WB	SB	WB	WB	WB	WB
		317–325	SB	SB	SB	SB	SB	SB	SB
		152–160	WB	WB	WB	WB	-	WB	WB
Pool IV	LPG-3	14–22	SB	WB	WB	WB	WB	SB	SB
		164–172	SB	SB	SB	WB	SB	SB	SB
		41–49	WB	SB	WB	WB	WB	WB	WB
		655–663	WB	SB	SB	WB	WB	SB	WB

*strong binder (Strong binders have an IC_50_ less than 50 nM).

**weak binder (Weak binders have an IC_50_ more than 50 nM and less than 500 nM.

### Study subjects

67 CL recovered individuals (28 men and 39 women ranging from 8 to 78 years old (25.3±16.8 years) were sampled from an endemic area for cutaneous leishmaniasis (Chaheshk, Mashhad suburbs, Khorasan province). The individuals had recovered from 4 months to 6 years ago (2.22±1.4 years) and had apparent scars (one or more) on their faces, hands and/or legs. All volunteers had recovered after a course of standard glucantime therapy. 66 healthy donors with no obvious signs and symptoms of leishmaniasis (60 men and 6 women) were also sampled from blood donation volunteers at Tehran Blood Transfusion Center ranging from 20 to 62 years old (40.4±11.3 year). All volunteers had donated blood more than once. 20 ml of blood was sampled in tubes containing sodium heparin (Rotexmedica, Germany) after signing an informed consent. 1 ml blood was also separately gathered in tubes containing 0.5 M EDTA (Merck, Germany) for DNA extraction.

### Ethics statement

The present study was approved by ethical committee of Pasteur Institute of Iran. All human blood samples were taken under supervision of internal medicine in both Tehran and Chaheshk (Mashhad) centers. All patients had signed the consent letter before sampling.

### Peripheral Blood Mononuclear Cell isolation

Peripheral Blood Mononuclear Cell (PBMC) was isolated by standard density gradient centrifugation protocol using Ficol-Hypaque. Briefly, 20 ml blood sample was diluted 1∶1 with sterile PBS buffer and uploaded on Ficol-Hypaque 1077 (Sigma, Germany) and centrifuged at room temperature for 40 min at 2200 rpm. Mononuclear layer was separated and washed with sterile PBS. Cells were counted and cryo-preserved in heat-inactivated Fetal Calf Serum (FCS)-10% DMSO until use.

### HLA-A2 typing

DNA from all samples was extracted by GF-1 Nucleic Acid Extraction Kit (Vivantis, Canada) according to the kit instructions. HLA-A2 positive individuals were screened by PCR-Sequence Specific Primer (PCR-SSP) method according to Bounce *et al.* with some modifications [71]. One pair of specific primers (P296 and P302) was used to detect HLA-A*0201-A*0217 alleles. One pair of HLA-DRB1 specific primers (P63 and P64) was used as internal control (Table 4). PCR reaction mixture contained: 1× PCR buffer (Amplisens biotechnologies, Moscow), 200 µM of each dNTP, 1.5 mM MgCl_2_, 0.04 u/µl *Taq* DNA polymerase (Amplisens biotechnologies, Moscow), 100 ng DNA, 1 pmol/µl each specific forward and reverse primers and 0.1 pmol/µl each forward and reverse internal control primers. The PCR cycling program consisted of 1 cycle of 94°C-4 min, 6 cycles of 94°C-25 sec, 65°C-45 sec. and 72°C-45 sec, 20 cycles of 94°C-25 sec, 61°C-45 sec. and 72°C-45 sec and a final extension of 72°C-2 min. The amplicon length was 489 bp and 796 bp for specific and internal control, respectively. T2 cells (ATCC CRL-1992) were used as PCR positive control. These cells are homozygout for HLA-A*0201 allele.

**Table 4:** Sequence Specific Primers used in PCR reactions for typing HLA-A2 and sub typing HLA-A*0201 allele.

Primers specificity	Primer	Sequence	Definition
HLA- A*0201-17 alleles	P296	GTG GAT AGA GCA GGA GGG	Forward specific primer
	P302	CCA AGA GCG CAG GTC CTCT	Reverse specific primer
	P63	TGC CAA GTG GAG CAC CCA A	Forward specific primer
	P64	GCA TCT TGC TCT GTG CAG AT	Reverse specific primer
	AL#37	CCT CGT CCC CAG GCT CT	Forward specific primer
HLA-A*0201 (1st PCR)	AL#AW	TGG CCC CTG GTA CCC GT	Reverse specific primer
	SG#A,	TGT CCG CCG CGG TCC AA	Forward specific primer
	SG#NA2	CTC GCC CCC AGG CTC C	Reverse specific primer
	SG#β2ms2	CGA TAT TCC TCA GGT ACT C	Forward specific primer
	SG#β2ma3	CAC AAC TTT CAG CAG CTT AC	Reverse specific primer
HLA-A*0201 (2nd PCR)	Al #22	CAC TCC ATG AGG TAT TTC TT	Forward specific primer
	AL #Q	CTC CAG GTA GGC TCT CAA	Reverse specific primer
	AL# BG	CGT CGC AGC CAT ACA TCC	Reverse specific primer
	AL# BF	CCC CAC GTC GCA GCC AT	Reverse specific primer
	Al# V	GAG CCA CTC CAC GCA CGT	Forward specific primer
	AL #14	AGG CCC ACT CAC AGA CTC	Reverse specific primer
	AL#3	GAC GGG GAG ACA CGG AAA	Reverse specific primer

### HLA-A*0201 typing

To screen HLA-A*0201 positive individuals, we used Gatz *et al.* nested PCR protocol [72] with minor modifications. For the first step, one pair of specific primer for HLA-A2 alleles (AL#37, AL#AW) and one pair for all other alleles except A2 (SG#A, and SG#NA2) were used. One pair of β2-microglobuline specific alleles (SG#β2ms2, and SG# β2ma3) was also used as internal control. The amplicon length was 812 bp for A2-specific reaction, 612 bp for non-A2-specific reaction, and 350 bp for internal control, respectively. For the second step, five sets of reactions were used: AL#22/AL#Q (718 bp), AL#22/AL#BF (547 bp), AL#22/AL#BG (542 bp), AL#V/AL#14 (543 bp) and AL#V/AL# 3 (565 bp). The sequence of primers is shown in Table 4. All PCR reactions contained, 1× PCR buffer, 200 µM each dNTP, 1.5 mM MgCl_2_, 0.04 u/µl *Taq* DNA polymerase,1.6 pmol/µl each specific primer and 0.4 pmol/µl each internal control, 100 ng DNA at first PCR reaction and 1 µl from 1∶5 diluted A2 PCR amplicon in the nested reaction. PCR cycling was set the same as before for A2 typing. If a sample was positive at first step (A2 positive), then the amplicon was transferred to the second step nested reaction. The sample was obviously HLA-A*0201 positive if all five sets of reactions were positive, although other A2 alleles may have also been present. If even one reaction was negative, the sample was HLA-A*0201 negative but other HLA-A2 alleles were present.

### *In vitro* stimulation of memory CD8^+^ T cells with predicted peptides

In this study, we used PBMCs from CL recovered individuals and healthy donors to assay the antigenicity of predicted peptides by short-term T cell clone preparation *in vitro*. For this, cryopreserved cells were thawed at standard condition. 2×10^6^ cells were suspended in 2 ml RPMI 1640 (Sigma, Germany) supplemented with 10% human AB serum (Sigma, Germany), 1% HEPES (Sigma, Germany), 2 mM L.Glutamine (Sigma, Germany) and 0.1% Gentamicin (Sigma, Germany), dispensed in 24-well culture plates (Orange Scientific, Switzerland) and incubated overnight at 37°C and 5% CO_2_ for rest. After 24 hours, cells were stimulated with peptide pools at 10 µg/ml/peptide and also freeze/thawed antigens of *L. major* (10 µg/ml) as indicator of previous disease. Recombinant human IL-2 (Sigma, Germany) at 10 ng/ml final concentration was added after 24 hours and every 3 days along the culture period. After 10 day culture cells were washed and re-stimulated at 96-well round bottom culture plates (Orange Scientific, Switzerland) with peptide pools at the same peptide concentration and anti-CD49d/anti-CD28 antibodies (BD biosciences) as co-stimulators at 1 µg/ml final concentration. Cells were also stimulated with Phorbol Myristate Acetate (50 ng/ml) (Sigma, Germany) and Ionomicin (500 ng/ml) (Sigma, Germany) mixture as control for cell activity and IFN-γ production. IFN-γ secretion was stopped by Berfeldin A (Sigma, Germany) at 20 µg/ml final concentration. After an overnight culture, cells were stained for surface markers (CD3 and CD8) and intracellular IFN-γ (all from BD biosciences) to be analyzed by flow cytometry. 1 ml of all supernatants was stored for further ELISA assay, before re-stimulation at day 10.

### Intracellular cytokine staining (ICCS) of IFN-γ producing cells

We referred to standard plate based protocols with some modifications [73], [74]. Briefly, after overnight incubation at 96-well round bottom culture plates, cells were pelleted, washed with PBS, and stained with anti-CD3-PE-Cy5 conjugated and anti-CD8-PE conjugated antibodies, for 30 min at 4°C. After complete washing, cells were fixed and permeabilized with Cytofix-Cytoperm buffer (BD biosciences) for 20 min at 4°C. After washing with buffer containing saponin (BD Cytofix-Cytoperm kit, BD biosciences), cells were stained with anti-IFN-γ-FITC conjugated antibody for 30 min at 4°C, then fixed in 1% Paraformaldehyde (Electron Microscopy Sciences, USA) solution. Freshly stained and fixed cells were analyzed with argon ion laser equipped BD FACScalibur flow cytometer (BD biosciences, USA). 250,000 events were collected and analyzed by FlowJo 7.5.3 (TreeStar, USA) for percent of CD8^+^/IFN-γ^+^ T cells in CD3^+^ region gated out of lymphocyte region.

### Enzyme Linked Immunosorbent Assay (ELISA) for secreted IFN-γ

Cytokine assay for IFN-γ production was performed according to Human IFN-γ ELISA kit instructions (R&D Systems, DuoSet, USA). After *in vitro* stimulation for 10 days, culture supernatants were collected and freezed. At the time of ELISA experiment, 150 µl of each culture supernatant (each in duplicate) was used to detect the specific IFN-γ production at related supernatants with kit instructions. IFN-γ concentrations were intercepted on the standard curve.

### Enzyme Linked Immunospot Assay (ELISpot) for IFN-γ and IL-4 secreting cells

ELISpot assay was followed as instructions provided in IFN-γ/IL-4 ELISpot kit (Diaclone, France) after *in vitro* stimulation for 10 days. PBMC were plated in duplicate at a concentration of 5×10^5^ cell/well along with peptide pools at a final concentration of 10 µg/ml/peptide. Spots were counted using a dissecting microscope (Nicon, Japan). Results were expressed as spot forming cells (SFC)/10^6^ PBMC. The sample was considered positive when the spots in stimulated wells were above mean+2SD of control un-stimulated wells of each sample.

### Statistical analysis

Fischer's exact test and non-parametrical Mann Whitney U tests were used to evaluate the difference between groups. *P* value less than 0.05 has been considered significant.

## Results

### *In silico* analysis of six known *L. major* proteins for potential candidate peptides

Through immunoinformatic analysis, eighteen 9-mer peptides were selected as HLA-A*0201 binders from six different proteins. Proteins were analyzed for best binding epitopes individually. The most important criteria were based on absolute scores from two mostly used online algorithms: SYFPEITHI with the score of more than 20 and BIMAS with the score of more than 100 (with some exceptions for BIMAS score if necessary). This first step limited the selected peptides from each protein matching both criteria to less than six peptides. In the next step of analysis, the screened peptides from the first step were more analyzed through five more algorithms to see whether the selected peptides were amongst high-ranked ones in each program. The prominent aspect of the selected algorithms is that they rank the peptides predicted out of a protein sequence according to absolute scores (or maximum binding score in Rankpep) and specify peptides as binders or non-binders based on a predetermined threshold. Peptides selected in the first step were adopted only if they passed the threshold of at least four algorithms of second step and sat at high rank peptide positions according to thresholds set for 80–85% accuracy. Finally, peptides selected through this sieving process were blasted with human and mice proteome and peptides with 100% identity were substituted if possible with others that had acceptable position passing through the sieving process. Table 2 summarizes the candidate epitopes characteristics. NetMHCPan1.1 algorithm predicted the selected peptides as binders to at least one more allele in the A2 supertype (Table 3). Peptides were pooled in four different groups, 4–5 peptides each: peptide pool I included CPB and CPC (5 peptides), peptide pool II included LmsTI-1 (4 peptides), peptide pool III included TSA and LeIF (5 peptides) and peptide pool IV included LPG-3 (4 peptides).

### HLA-A2 typing

**Figure 1:**
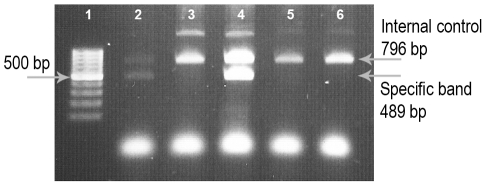
HLA-A2 screening by one step PCR. One step PCR-SSP method was used to screen for HLA-A2 positives among all samples included (recovered individuals and healthy donors). Lane 1 shows 100 bp DNA ladder marker, lane 4 shows the PCR reaction of T2 cells as positive control, lane 3, 5 and 6 is related to negative samples and lane 2 is related to a positive sample.

Using a well designed PCR-SSP method, we were able to screen 26 and 22 A2 positive individuals out of 67 *L. major* recovered (38%) and 66 healthy donors (33%), respectively. Of these, 19 HLA-A2^+^ recovered individuals were included in the test group, 11 HLA-A2^−^ recovered individuals were selected as control group to define the specificity of *in vitro* evaluated peptides for HLA-A2 and 6 HLA-A2^+^ healthy donors were selected as control group to define the specificity of *in vitro* evaluated peptides for *L. major*. 15 out of 19 HLA-A2^+^ recovered individuals (78%) and 5 out of 6 healthy donors (83%) were HLA-A*0201 positive, respectively. Figure 1 shows the PCR amplicon of T2 cells, HLA-A2^+^ and HLA-A2^−^ samples.

### Specific memory CD8^+^ T cells are detectable in PBMC of cutaneous leishmaniasis recovered individuals

**Figure 2:**
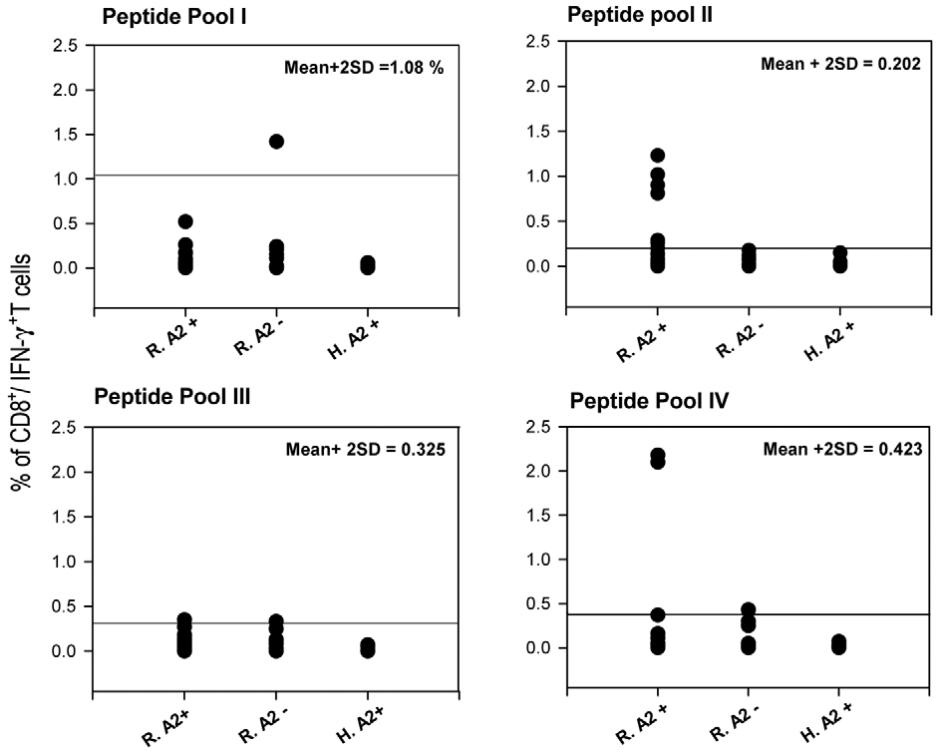
Status of CD8^+^/IFN-γ^+^ T cell response of recovered HLA-A2^+^ individuals compared to HLA-A2^−^ individuals. 6 out of 19 (31.6%) and 2 out of 15 (13.3%) HLA-A2^+^ recovered individuals responded above cutoff value (horizontal bar in each plot defined as mean + 2SD CD8^+^/IFN-γ^+^ response in HLA-A2^−^ controls) against peptide pools II and IV, respectively. Fischer's exact probability test showed that the response in peptide pool II is statistically significant.

**Figure 3:**
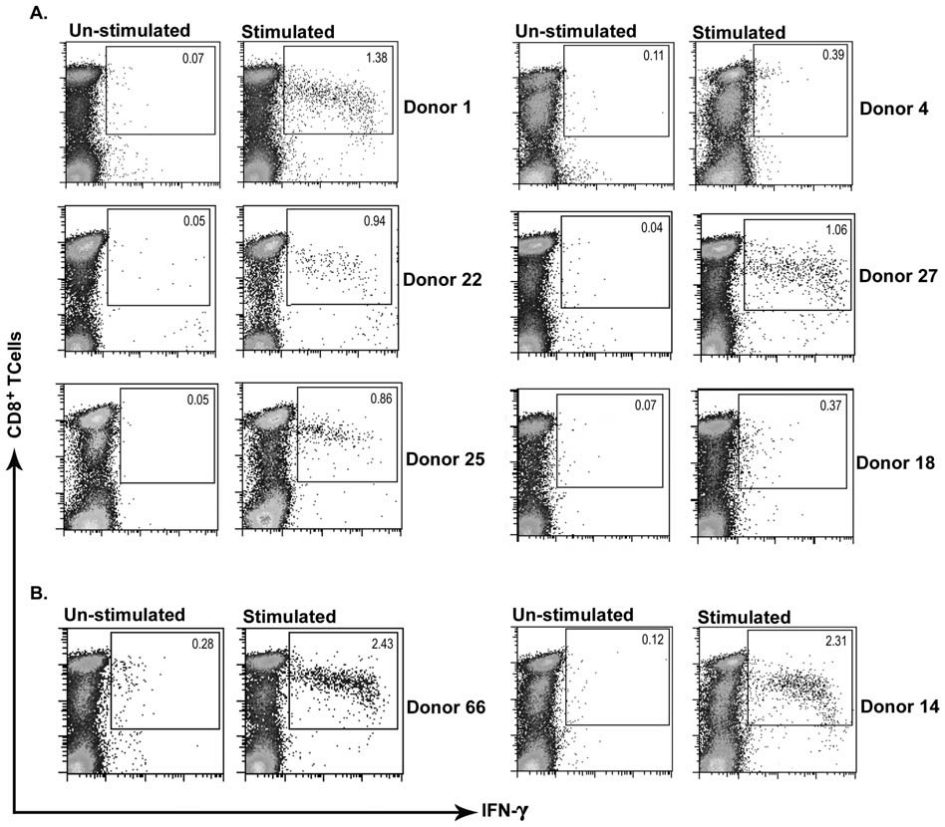
Flow cytometric analysis of IFN-γ production in positive samples. Positive response of six HLA-A2^+^ individuals against peptides of pool II (A) and 2 HLA-A2^+^ individuals against peptides of pool IV (B) is depicted versus un-stimulated control of each sample. Dot plots show CD8 vs. IFN-γ staining. Upper right squares define the CD8^+^/IFN-γ^+^ region. Numbers represent the percentage of IFN-γ producing CD3^+^/CD8^+^ T cells in the lymphocyte gate.

**Figure 4:**
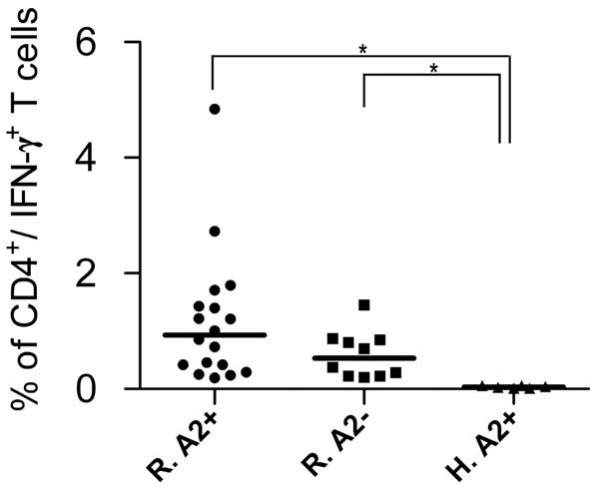
The response of individual volunteers to *L. major* lysate. Freezed/thawed antigens of *L. major* were used to stimulate the PBMCs in culture as previous disease indicator. The responses of *L. major* recovered individuals were potentially detected at CD4 level. Each point represents response of each individual. Horizontal bars represent the median value of CD4^+^/IFN γ^+^ T cells in the related group. Statistical analysis shows a significant difference between R.A2^+^ (HLA-A2^+^ recovered individuals) and R.A2^−^ (HLA-A2^−^ recovered individuals) groups with H.A2^+^ individuals (HLA-A2^+^ healthy donors) with *p* value<0.05.

To assay the antigenicity of predicted peptides *in vitro*, short-term T cell clone preparation was used. Although short-term culture stimulation next to peptide (6–12 hrs long) followed by ICCS is introduced as a potent CD8^+^ T cell detecting system [74], [75], we used an *in vitro* stimulation protocol before ICCS to more sensitively detect the very low frequency response of the CL recovered individuals that had recovered years ago. Individual response of HLA-A2^+^ recovered volunteers (represented by percent of CD8^+^/IFN-γ^+^ T cells) after *in vitro* stimulation against peptide pools II (19 samples tested) was notably higher than that of HLA-A2^−^ recovered individuals (*p* value = 0.03 by Mann Whitney U test) and healthy donors (*p* value = 0.036 by Mann Whitney U test). In contrast, Individual response of HLA-A2^+^ recovered volunteers against other peptide pools, I and III and IV (15 samples tested each) were not statistically significant compared to the response of HLA-A2^−^ recovered individuals (*p* value>0.05), but were significant against healthy control donors (*p* value<0.05). HLA-A2^−^ recovered individuals were included in this study to show the specificity of response to HLA-A2 alleles. HLA-A2^+^ healthy donors were included to show the specificity of response to *L. major* epitopes. No response was totally detected in this latter control group against any peptide pool stimulation. We used the mean+2 SD of CD8^+^/IFN-γ^+^ T cells in percent in HLA-A2^−^ individuals as the cutoff for categorizing individuals in both HLA-A2^+^ and HLA-A2^−^ groups to responders and non-responders and find the association between these two categories (Figure 2). 6 out of 19 (31.6%) HLA-A2^+^ recovered ones responded above cutoff values (mean+2 SD = 0.2) in peptide pool II statistically significant by Fisher's exact probability test (*p* value = 0.045). 2 out of 15 (13.3%) HLA-A2^+^ recovered ones responded notably higher than cutoff (mean+2 SD = 0.42) in peptide pool IV in comparison to HLA-A2^−^ individuals (Figure 2), but this was not statistically significant (*p* value>0.05). In the next two peptide pools, the HLA-A2^−^ recovered individuals showed positive responses as high as HLA-A2^+^ individuals (peptide pool III) or even higher (peptide pool I), which renders the difference statistically not significant by Fisher's exact probability test (*p* value>0.05). However, this recommends that the predicted peptides may act as promiscuous epitopes, able to bind to a wider range of HLA alleles besides HLA-A2, and this is noteworthy to be further studied. Figure 3 depicts the positive response in six HLA-A2^+^ responders against peptide pool II and the positive response of two HLA-A2^+^ responders against peptide pool IV. Excitingly, all responders were typed as HLA-A*0201 except one which was typed as HLA-A*0202 or HLA-A*0203. This sample showed lower response than most of the other responders who were HLA-A*0201 positive (Figure 3, Donor 4). The response of volunteers to *L. major* lysate (freezed/thawed antigens of parasite) was potentially detected at CD4 level and was used as indicator of previous disease to assure previous infection and is summarized in Figure 4. As shown in this figure, the difference between HLA-A2^+^ recovered individuals and HLA-A2^−^ individuals is not significant (*p* value = 0.054, Mann-Whitney U test) but the difference between HLA-A2^+^ recovered individuals and healthy donors (*p* value = 0.0002, Mann-Whitney U test) and between HLA-A2^−^ recovered individuals and healthy donors (*p* value = 0.0007, Mann-Whitney U test) is significant with *p* value<0.05. This confirmed the previous infection in recovered individuals and no infection in healthy donors.

**Figure 5:**
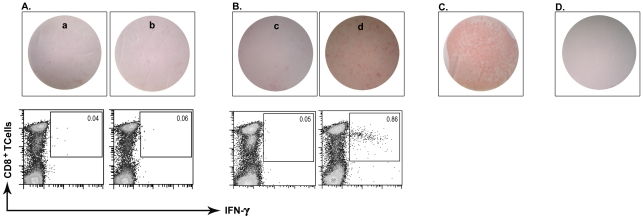
Enumeration of peptide-specific IFN-γ producing T cells stimulated against peptide pool II by ELISpot assay. ELISpot analysis (upper row) of a negative responder (HLA-A2^−^ recovered individual) (A) and a positive responder (HLA-A2^+^ recovered individual) (B) against peptide pool II stimulation is depicted compared to flow cytometric results (lower row) of the same samples. PMA/Ion stimulation (C) and culture medium only (D) are used as positive and negative controls, respectively. (a and c are related to un-stimulated controls of each sample, b and d are related to stimulated samples with peptide pool II). Dot plots show CD8 vs. IFN-γ staining. Upper right squares in each plot define the CD8^+^/IFN-γ^+^ region. Numbers represent the percentage of IFN-γ producing CD3^+^/CD8^+^ T cells in the lymphocyte gate.

To further confirm the ICCS results, peptide-specific IFN-γ producing T cells were enumerated via ELISpot assay. Although ELISpot is often performed without any preceding *in vitro* stimulation, we performed exactly the same as previous to make the results comparable to ICCS. Cells from three HLA-A2^+^ responders to peptide pool II and one HLA-A2^−^ non-responder individual were stimulated *in vitro* against peptide pool II (as described in materials and methods). After 10 days, they were re-stimulated next to relevant peptides in 96-well PVDF-bottomed plates coated with anti-human IFN-γ and anti-human IL-4 antibodies for 48 hrs. Figure 5 depicts the positive response of one of the responders that had detectable amount of IFN-γ production against peptide pool II (101 SFC/10^6^ PBMC in stimulated wells vs. 60 SFC/10^6^ PBMC in un-stimulated wells). No IL-4 was detected in this sample. On the other hand, HLA-A2^−^ non-responder individual showed no detectable response. (13 SFC/10^6^ PBMC in stimulated wells vs. 42 SFC/10^6^ PBMC in un-stimulated wells). Even though the other two responders had no detectable response in ELISpot, this could be explained by the higher sensitivity of ICCS to ELISpot proposed by some other researchers [76], [77].

### Secreted IFN-γ from PBMCs of HLA-A2^+^ recovered individuals stimulated against peptide pool II and IV is detectable by ELISA in culture supernatants

**Figure 6:**
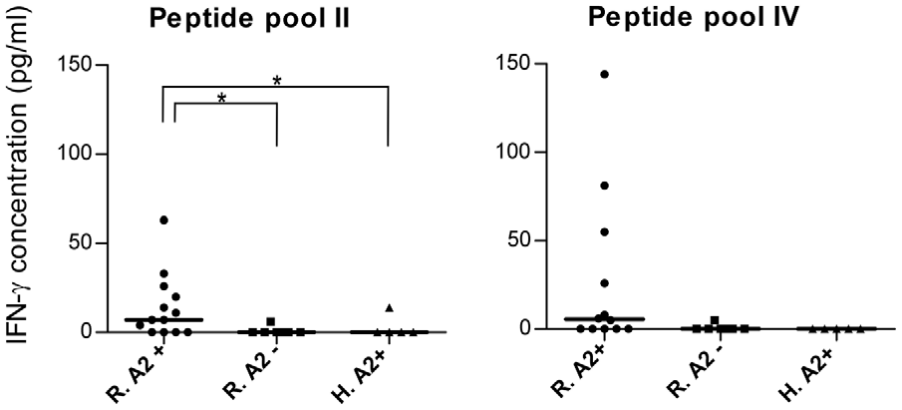
Detection of secreted IFN-γ from PBMCs stimulated against peptide pool II and IV by ELISA in culture supernatants. IFN-γ production was measured in culture supernatants of HLA-A2^+^ recovered individuals (R.A2^+^), HLA-A2^−^ recovered individuals (R.A2^−^), and HLA-A2^+^ healthy donors (H.A2^+^) stimulated against peptide pools II and IV. Each point represents the net result of individual experiments. Horizontal bars represent the median value of the IFN-γ concentration in the related group. The response of R.A2^+^ individuals was detected higher than that of R.A2^−^ ones in peptide pools II and IV.

Since we had detected HLA-A2 specific positive responses in peptide pool II and IV, we used an ELISA system (with a sensitivity of 15 pg/ml) to check the cytokine release in the supernatants of cells cultured next to these peptide groups. Culture supernatants of peptide pools II and IV stimulated samples were analyzed for IFN-γ production on day 10 before re-stimulation. By intercepting all OD data on a standard curve, IFN-γ concentration was calculated in non-stimulated wells as background and also stimulated wells. Net production was subtraction result of background (un-stimulated wells) from stimulated wells. As shown in Figure 6, IFN-γ production against peptide pool II stimulation was significantly higher in HLA-A2^+^ recovered individuals compared to HLA-A2^−^ recovered ones (*p* value = 0.011, Mann Whitney U test) and healthy donors (*p* value = 0.031, Mann Whitney U test). Although higher responses were detected in peptide pool IV, the difference was not statistically significant. We used the mean+2 SD of IFN-γ concentration in HLA-A2^−^ individuals as the cutoff for categorizing individuals in both HLA-A2^+^ and HLA-A2^−^ groups to responders and non-responders and find the association between these two categories. 8 out of 13 (61.5%) and 4 out of 12 (33%) HLA-A2^+^ recovered individuals had detectable cytokine production well above the cutoff scores. Fisher's exact probability test showed significant difference in peptide pool II (*p* value = 0.0063) but not in peptide pool IV. Although ELISA never ascribes the cytokine production to a specific cell type when whole PBMC is in use, but it is indicated that 9-mer peptides as recall antigen are only able to induce the IFN-γ production from CD8^+^ T cells. Therefore, totally these results are well in concordance with previous results from flow cytometry analysis. The response in peptide pool IV may need to be further analyzed in a larger group of patients to statistically turn significant, but couldn't be neglected due to main purpose of this part of the study which was to identify the peptides naturally processed and presented after infection, among all predicted ones.

## Discussion

CD8^+^ T cells primed at Th1 milieu can participate in immune response by IFN-γ production to activate macrophages, by cytotoxic activity via perforin/granzyme production and/or Fas-Fas ligand interaction or both mechanisms to disrupt the parasite host [78]. One of the most effective strategies to activate CD8^+^ T cell responses while avoiding whole parasite structure as in leishmanization or deleterious sequences that can activate harmful Th2 responses as in protein subunit vaccines is “Polytope Vaccine” strategy. Multiple CD8^+^ T cell activating epitopes could be included in the vaccine construct to induce a multi-CD8^+^ T cell response [79], [80].

There are very few reports about *Leishmania* specific CD8^+^ epitope mapping. Therefore, to begin for the polytope strategy, massive experiments need to be conducted to map the promising epitopes from different proteins of *Leishmania* species. Controversy still remains regarding the route of activation of CD8^+^ T cells in leishmaniasis, since *Leishmania* resides within parasitophorous vacuoles of the macrophage and it is not clear how these cells present *Leishmania* antigens to CD8^+^ T cells through MHC class I [78]. Part of the existing data suggests that only external or secreted *Leishmania* antigens are able to reach macrophage cytosol to be presented in the context of HLA class I molecules, which is a prerequisite for CD8^+^ T cell activation [81], [82]. However, Requena *et al.* have shown specific CD8^+^ T cell responses against nucleosomal histones which are non-secretory proteins [49]. This makes all the proteins noteworthy to be evaluated for CD8^+^ T cell epitopes. We focused on proteins with reported CD8^+^ T cell response in literature and acceptable level of expression in amastigote stage.

To speed up the mapping process, immunoinformatic tools are now in access. Plenty of algorithms are available on World Wide Web [83]–[85], but there is no consensus on the most appropriate one to predict more true positive epitopes. Due to lack of a consensus mapping protocol with immunoinformatic tools, we based our prediction on Trost *et al.* theory that greater prediction accuracy can be achieved by combining the predictions from several algorithms rather than relying on just one [86]. So we started the prediction with two quantitative matrix methods most-commonly in use: SYFPEITHI and BIMAS. These methods do not differentiate binders and non-binders and one should practically evaluate the immunogenicity of about 10% of top scored peptides of a protein to mark positives with 85% accuracy [87]. This is simplified by choosing peptides with scores higher than 20 and 100 for SYFPEITHI and BIMAS, respectively. It is believed that peptides selected on these scores are energetically best fitted to HLA peptide binding groove [88]–[93]. It must be noted that BIMAS scores are sometimes disappointing and do not catch up to ideal score. This is due to an innate characteristic of the algorithm that considers each amino acid's effect on overall affinity by its own and regardless of other amino acids in the peptide sequence [85]. For this reason we did not expect BIMAS score to be ideal in some cases. Although a protein may have more than 30 peptides scoring above 20 at SYFPEITHI, these are restricted to very few epitopes scoring above 100 at BIMAS. This was a limiting step reducing peptides to be analyzed for each protein to less than 6.

At the second step of analysis, peptides were further evaluated with EpiJen, Rankpep, nHLApred, NetCTL and Multipred. These models are trained on binder and non-binder data and discriminate these two based on predetermined thresholds. Peptides selected at the first step were further approved only if passed the threshold of at least four algorithms at the second step of analysis. It should be mentioned that we did not select the peptides according to their ranks in these algorithms because it is not a consensus yet that peptides with higher rank predicted by different algorithms (within those top scores beyond thresholds of binding) are absolutely binders [94]. Different algorithms propose that the peptides with scores (either absolute or maximum binding) above the thresholds (setting to 80–85% accuracy) are top-score peptides and one should test them all to find a binder *in vitro* and *in vivo*.

HLA molecules that present the epitopes to the T cells are among the most polymorphic molecules in human populations, so the vaccine composing epitopes must cover the population HLA diversity. The concept of HLA supertypes helps to reduce the number of epitopes needed for this purpose, since many HLA alleles, although different in sequence, have common binding specificities. They can bind a peptide with more or less identical affinity and are clustered in a defined supertype as A2, A3 and so on [95], [96]. These so-called promiscuous T cell epitopes that bind several alleles at a supertype or between different supertypes are advantageous for maximal population coverage [97], [98]. NetMHCpan1.1 specified that selected peptides were binders to at least one more HLA-A2 supertype alleles.

All prediction algorithms used in this project are indirect methods that predict HLA binding, though not all HLA binders are T cell epitopes [99]. *In silico* analysis even with high sensitivity and specificity is just a prediction. There are no consensus ways to predict desirable peptides with 100% accuracy. This necessitates *in vitro* and *in vivo* evaluations to confirm antigenicity [100], [101]. *In vitro* stimulation to recall memory CD8^+^ T cells from *Leishmania*-infected individuals and intracellular cytokine assay for IFN-γ producing cells confirmed that HLA-A2^+^ CL recovered individuals have developed specific response against peptides from LmsTI-1 and LPG-3 during the active phase of their disease. This is of paramount importance since it shows that the peptides are real T cell epitopes naturally processed and presented to immune system during *Leishmania* infection. It might even have been better to screen CD8^+^ T cell response in asymptomatic (sub-clinical) individuals since these are naturally resistant individuals who may have established stronger immune responses against leishmanial antigens. This study also showed that the prediction is almost reliable by the algorithm combination we used. Chentoufi *et al.* previously showed that a combination of SYFPEITHI and BIMAS considering proteasomal cleavage predicted by NetCHop, MHC pathway and MAPPP accurately specifies three potent epitopes out of ten predicted ones from one protein [102]. Although we have not dissected the peptide pools to individual peptides responsible for the positive response (because of a small lymphocyte source and also difficulty to discriminate a positive response against an individual peptide due to time elapsed after recovery (averagely two years), obviously at least two peptides (one from each pool of II and IV) are qualified out of the eighteen predicted ones. This confirms that QM algorithms in combination with machine learning methods and ANN in particular can confine the peptides to a small group worthwhile to be focused on *ex vivo*. The results of this study showed that the way we predicted peptides could discriminate probably dominant epitopes out of candidate proteins.

According to HLA–A*0201 typing results, 83% of HLA-A2^+^ responders to pool II (5 out of 6) and both HLA-A2^+^ responders to pool IV are HLA-A*0201 positive. One of the responders to peptide pool II, was HLA-A*0201 negative .This is consistent with promiscuity that predicted peptides could be presented in HLA-A2 alleles other than HLA-A*0201, and needs to be further confirmed in a larger population of HLA-A2 individuals bearing other HLA-A2 alleles. 76% of HLA-A2^+^ recovered non-responder individuals to peptide pool II (10 out of 13) and 84% of non-responders to peptide pool IV (11 out of 13) were HLA- A*0201 positive. Although only 31.6% and 13.3% of CL recovered HLA-A2^+^ individuals responded in Peptide pools II and IV, respectively, this is expectable and in complete agreement with other studies evaluating peptide immunogenicity by recall responses from infected individuals [90]. It might be a question whether pooling the peptides adversely affects the response of each individual peptide, but this seems unlikely since we have detected response in all peptide pools. Therefore, pooling peptides does not seem a limiting factor [103], [104] considering that all peptides are predicted with almost equal affinity for HLA binding and with same stimulatory concentration at cultures. Of course this remains to be more elucidated in an *in vivo* assay with HLA-A2 transgenic mice. Other factors could be responsible in this regard including: 1- individual TCR repertoire, 2- stimulation conditions *in vitro* which was set up the same way for all samples (based on the immune response potency in each individual, some samples may need more rigorous *in vitro* stimulation), 3- the time elapsed after recovery that is different between individuals and affects the frequency of existing memory T cells at blood samples, 4- actual protein expression and processing level and 5- the most important of all, the HLA content of every individual. The HLA content may direct the response toward non-A2 alleles that potentially present other peptides than predicted ones during natural course of the disease.

In this study, we also detected positive responses in HLA-A2^−^ recovered individuals against other two protein groups (CPB, CPC, LeIF and TSA), which are also among the best candidate antigens for vaccine design. It might be questioned how peptides bearing HLA-A2 super-motif binding specificities bind other alleles of different supertypes. One possible explanation could be the overlap between supertypes in terms of specificity that a peptide binds alleles in other supertypes [96]. So it is noteworthy to further analyze these peptides for their HLA restrictions.

This is a novel study recording *L. major* specific CD8^+^ T cell responses against peptides presented in the context of human HLA. The only recent report refers back to Walden *et al.* that mapped potential T cell epitopes from Kinetoplastid membrane protein of *L. major* (Kmp-11) via classical mapping for different human HLA class I alleles [47]. Gazzinelli *et al.* have studied CD8^+^ T cell responses against *L. donovani* A2 specific MHC I binding peptide determined by BIMAS and have demonstrated that A2-specific T cell responses are responsible for reduced parasitism in both liver and spleen of BALB/c mice immunized with A2 and challenged with *L. chagasi* [52]. Instead of one protein at a time, Laouini *et al.* and Dumonteil *et al.* started genome-wide screenings for novel epitopes in separate labs. This approach is for certain impossible via classical mapping. However, using a combination of T cell epitope prediction tools (QMs and machine learning methods); both groups have successfully validated epitopes in BALB/c mice [94], [105]. These studies have put forward novel candidate antigens for vaccine development not previously reported. The peptides selected in this study are highly conserved among different species of *Leishmania* as *L. tropica*, *L. donovani*, *L. mexicana* and *L. braziliensis* and even other kinetoplastids. This sparks the idea of multi-species specific vaccine at first look especially for visceral leishmaniasis, in which CD8^+^ T cell activation is a must. However, this should be considered with percussions about other cases such as muco-cutaneous leishmaniasis that progress through an uncontrolled immune response.

Today, *in Silico* prediction studies could be complemented easily by testing the immunogenicity of the predicted epitopes in HLA transgenic mice bearing human HLA molecules instead of their own MHC class I molecules [106]. This is a shortcut through peptide evaluation for human polytope vaccines and there is no need to predict for mouse-specific peptides. Our next approach is to design a DNA construct based on the peptides related to LmsTI-1 and LPG-3 and even the peptides from CPB and CPC proteins and immunize HLA-A2 transgenic mice bearing HLA-A*0201 allele to follow the immune response against individual peptides *in vivo*. We believe that *in vivo* stimulation will give better responses than *in vitro* stimulation so we can clearly dissect the individual peptides of each pool responsible for positive responses. It is necessary to assess the cytotoxic activity of stimulated CD8^+^ T cell clones since these clones could act in an unpredictable way when triggered at very beginning of the response, and may undesirably lead to pathogenesis administered as a vaccine regimen. It would be useful to challenge the immunized mice with infectious *L. major* to check for the potency of this polytope structure to protect the mice against infectious *Leishmania* parasite.
